# The proteorhodopsins of the dinoflagellate *Oxyrrhis marina*: ultrastructure and localization by immunofluorescence light microscopy and immunoelectron microscopy

**DOI:** 10.1007/s00709-020-01530-z

**Published:** 2020-07-03

**Authors:** Erhard Rhiel, Martin Westermann, Frank Steiniger, Christian Hoischen

**Affiliations:** 1 Planktology, Institute for Chemistry and Biology of the Marine Environment, Carl von Ossietzky University Oldenburg, Carl-von-Ossietzky-Straße 9–11, 26111 Oldenburg, Germany; 2 Planktology, ICBM, Carl von Ossietzky University Oldenburg, P. O. B. 2503, 26129 Oldenburg, Germany; 3 Electron Microscopy Center at the Jena University Hospital, Friedrich-Schiller-University Jena, Ziegelmühlenweg 1, 07743 Jena, Germany; 4 CF Imaging, Leipniz Institute on Aging, Fritz-Lipmann-Institute (FLI), Beutenbergstraße 11, 07745 Jena, Germany

**Keywords:** Dinoflagellate, Immunofluorescence light microscopy, Freeze-fracture immunolabeling, *Oxyrrhis marina*, Proteorhodopsin, Transmission electron microscopy

## Abstract

At least 7 proteorhodopsin sequences of *Oxyrrhis marina* were recently proven in bands obtained by sucrose density gradient centrifugation, and MS analyses revealed that the bands consisted almost of pure, native proteorhodopsins (Rhiel et al. 2020). The proteorhodopsin fractions, i.e., bands B2, B3, and B4 were subjected to transmission electron microscopy. Negative staining revealed that band B2 consisted most likely of monomeric/oligomeric proteorhodopsins with particle dimensions of about 6 nm. Negative staining, freeze-fracture, and cryo-transmission electron microscopy revealed that bands B3 and B4 consisted of vesicular, sheet-like, and cup-shaped structures which all seemed to be composed of protein. Frequently, ring-like protein aggregates were registered at higher magnifications. They measured about 4 nm in diameter with a tiny hole of 1.5 nm in the middle. The bands B2, B3, and B4 were pooled and used to raise an antiserum. Immunoelectron microscopy resulted in intense labeling of the isolated structures. Immunofluorescence light microscopy of formaldehyde-fixed *Oxyrrhis* cells resulted in intense labeling of the cell periphery. Some cell internal structures became labeled, too. Immunoelectron microscopy of freeze-fractured cells revealed that most likely the membranes of the amphiesmal vesicles were labeled at the cell periphery, while the cell internal label seemed to originate from the food vacuoles.

## Introduction

Proteorhodopsins were first identified in the course of a metagenomic screening of uncultured sea samples from the Monterey Bay in California (SAR86). The name refers to its initial identification in the group of γ-proteobacteria, but meanwhile proteorhodopsins were proven for α-, β-, and δ-proteobacteria, bacteroidetes, chloroflexi, *Deinococcus-Thermus*, flavobacteria, firmicutes, planctomycetes, and actinobacteria as well. Proteorhodopsins were also detected in archaea (Euryarchaea) and in eukaryotic marine protists (Bamann et al. 2014; DeLong and Béjà 2010), with the first report of proteorhodopsin in protists presented by Lin et al. (2010). With respect to protists, proteorhodopsin-like genes have been identified in diatoms (*Pseudo-nitzschia granii*), haptophytes (*Chrysochromulina tobin*, *Phaeocystis antarctica*, *Phaeocystis globosa*, *Pleurochrysis carterae*, and *Prymnesium polylepis*), cryptophytes (*Chroomonas mesostigmata*, *Guillardia theta*, and *Hemiselmis andersenii*), and dinoflagellates (*Alexandrium catenella*, *Oxyrrhis marina*, *Polarella glacialis*, *Prorocentrum donghaiense*, *Prorocentrum minimum*, and *Pyrocystis lunula*) (Guo et al. 2014; Hovde et al. 2015; Li et al. 2019; Lin et al. 2010; Marchetti et al. 2015; Shi et al. 2015; Slamovits et al. 2011, and references cited therein).

For *Oxyrrhis marina*, 18 proteorhodopsin sequences are deposited at the NCBI databases. The proteins are 214–264 amino acids in lengths and show molecular weights of 23–29 kDa. The lower molecular weights are most likely due to incomplete sequences. Typical for proteorhodopsins, all of them are characterized by seven transmembrane spanning helices, and all except protein ABV22430, whose amino acid sequence is incomplete, show acidic pI values. In a previous study, fractions consisting of almost pure, native proteorhodopsins of *Oxyrrhis* were obtained by sucrose density gradient centrifugation. They were subjected to sodium dodecyl sulfate polyacrylamide gel electrophoresis. Subsequent mass spectrometry (MS) confirmed that in total at least 7 proteorhodopsins were present in three of the four sucrose bands, i.e., bands B2, B3, and B4 (Rhiel et al. 2020).

The current study continues on these results and focuses first on the ultrastructure of the sucrose bands. Here, negative staining, freeze-fracture, and cryo-transmission electron microscopy (Cryo-TEM) were applied. Second, an antiserum against proteorhodopsins was raised in rabbit and tested by western immunoblotting. Third, immunoelectron microscopy was applied to the sucrose band B3 as it most likely was constituted by aggregated proteorhodopsins. Fourth, immunofluorescence light microscopy and immunoelectron microscopy of freeze-fractured cells were conducted in order to gain information on localization of proteorhodopsins within *Oxyrrhis* cells.

## Materials and methods

### Strain sources and growth conditions

*O. marina* was obtained from the Culture Collection of Algae at Göttingen University (strain B21.89; SAG, University of Göttingen, Germany) and was cultured in f/2 medium (Guillard and Ryther 1962) as described by Nguyen et al. (2019). Mainly, cultures grown for 1 week in fresh medium with yeast cells as prey were used for the preparations of native proteorhodopsin fractions. Cells of a small-sized diatom were present in the *Oxyrrhis* cultures in tiny amounts and preyed as well (Rhiel 2017).

### Preparations of native proteorhodopsin fractions

Fractions, i.e., bands of native proteorhodopsins of *Oxyrrhis*, were obtained by sucrose density gradient centrifugation as described by Rhiel et al. (2020). The bands were withdrawn from the centrifuge tubes with either a syringe or a pipette and concentrated by diluting them with distilled water, followed by ultracentrifugation for 20 h at 8 °C, using a 70 Ti-fixed angle rotor and a L8-55M ultracentrifuge (Beckman Coulter, Krefeld, Germany). The pelleted bands were dissolved in 50-μl distilled water. Aliquots of those proteorhodopsin fractions were used for negative staining transmission electron microscopy, freeze-fracture transmission electron microscopy, Cryo-TEM, immunoelectron microscopy, SDS-PAGE and western immunoblotting, and for antiserum production.

### SDS-PAGE

SDS-PAGE was performed as described by Bathke et al. (1999). Aliquots (generally 50 μl) of the proteorhodopsin fractions were mixed with loading buffer containing either 50 mM dithiothreitol or 5% (v/v) ß-mercaptoethanol, heated for 5 min at 95 °C, and loaded onto 15% polyacrylamide gels using the buffer system of Laemmli (1970). The gels were run at constant voltage of 20 V overnight and afterwards stained with Serva Blue R (0.5%, w/v) dissolved in distilled water/methanol/acetic acid (5:5:1). Pictures of Coomassie-stained gels were taken with an Olympus C3030 Zoom digital still CCD camera.

### Antiserum production

The sucrose bands (i.e., bands B2, B3, and B4) from several experiments were pooled, diluted with distilled water, and concentrated by centrifugation as described by Rhiel et al. (2020). The resulting pelleted protein fraction, showing only one protein of 25 kDa was used for immunization. The immunization of a rabbit and subsequent antiserum collection was done by a commercial facility (BioScience, Göttingen, Germany). Preimmune serum was taken before the first immunization. Two weeks after the final immunization, the antiserum was obtained.

### Western immunoblotting

The specificity of the antiserum directed against the pooled proteorhodopsin fractions was controlled by subjecting those fractions to SDS-PAGE followed by western immunoblotting onto Protran BA 85 nitrocellulose transfer membrane (pore size 0.45 μm, Schleicher & Schüll GmbH, Dassel, Germany) according to Towbin et al. (1979). Preimmune serum and antiserum were used in dilutions of 1:200 up to 1:2000. Pictures of western immunoblots were taken with an Olympus C3030 Zoom digital still CCD camera.

### Negative staining and freeze-fracture transmission electron microscopy

Negative staining and freeze-fracture of the proteorhodopsin fractions were performed as described by Rhiel and Westermann (2012). Samples were examined with a digital Zeiss EM 900 electron microscope (Zeiss, Oberkochen, Germany; digital upgrade by Point Electronic, Halle, Germany) operated at 80 kV. Digitized images were taken with a wide-angle dual speed 2 K CCD camera controlled by the Sharp:Eye base controller and operated by the Image SP software (TRS, Moorenweis, Germany).

### Cryo-transmission electron microscopy

Cryo-TEM was performed as described earlier (Rhiel et al. 2013). Briefly, a small droplet (5 μl) of the proteorhodopsin fraction, i.e., band B3 was placed on a copper grid covered by a holey carbon film (Quantifoil R 1.2/1.3, pore size 1.2 μm, 400 mesh; Quantifoil Micro Tools, Jena, Germany). Excess liquid was blotted for 3 s between two strips of filter paper. Subsequently, the sample was rapidly plunged into liquid ethane (cooled to about − 180 °C with liquid nitrogen) in a Cryobox (Zeiss, Oberkochen, Germany). The frozen specimen was transferred with a cryo-holder (Gatan 626-DH, Gatan, Pleasanton, USA) into a precooled cryo-transmission electron microscope (CM 120, FEI, Eindhoven, Netherlands) operated at 120 kV and viewed under low dose conditions. Cryo-TEM images were recorded with a 2 K CMOS Camera (F216, software EMMENU V4.0; camera and software TVIPS, Munich, Germany).

### Immunoelectron microscopy of proteorhodopsin fraction B3

For immunoelectron microscopy followed by negative staining, the proteorhodopsin fraction, i.e., band B3, was allowed to adsorb for 5 to 10 min onto glow discharge treated carbon-coated grids. Then, the grids were floated for 1 h onto 50-μl drops of 1% of BSA dissolved in TBS, incubated for 1 h on 50-μl drops of either 1% of BSA dissolved in TBS or antiserum. The antiserum was diluted 1:100 in TBS containing 0.1% BSA. Then, the grids were washed in 10 drops of TBS and incubated for 1 h in gold-labeled goat anti-rabbit IgG conjugate (British Biocell International, Cardiff, UK, diluted 1:50 in TBS containing 1% BSA, 10 nm gold particles). After washing with 10 drops of TBS, the grids were stained with 1% (w/v) uranyl acetate dissolved in distilled water and used for transmission electron microscopy.

### Immunofluorescence light microscopy of cells of *Oxyrrhis marina*

For immunofluorescence light microscopy, *Oxyrrhis* cells from 100 to 150 ml of culture volume were harvested by centrifugation at 900×*g* for 10 min in an Eppendorf 5810R refrigerating centrifuge equipped with an A-4-62 swinging bucket rotor (Eppendorf, Hamburg, Germany), and resuspended in 1 ml f/2 medium. Fixation was achieved by adding an equal volume of 8% (v/v) formaldehyde dissolved in f/2 medium to a final concentration of 4%. After an incubation time of 1 h at room temperature, the cells were washed in 20 mM Tris–HCl pH 7.5, 150 mM NaCl (TBS) twice for 5 min each. Permeabilization of the cells was achieved by incubating them for 30 min in TBS containing 0.1% (v/v) Triton X-100. After two washings with TBS, the cells were incubated for 2 h at room temperature in 2% (w/v) of bovine serum albumin (BSA) dissolved in TBS. Then, the cells were kept for overnight at 4 °C in either 2% BSA dissolved in TBS (negative control) or incubated with antiserum diluted 1:10 in TBS containing 2% BSA. Afterwards, the cells were washed twice with TBS, and further incubated for 1 h at room temperature in Alexa Fluor 488-labeled goat anti rabbit IgG (H + L) conjugate (Invitrogen, Thermo Fisher Scientific, Waltham, USA) diluted 1:1000 in TBS containing 2% BSA. Then, the cells were washed twice in TBS and used for immunofluorescence light microscopy.

For an epifluorescence light microscopy, initially a Zeiss Axioskop 2 (Zeiss, Oberkochen, Germany) equipped with a 63-fold Zeiss Plan-Neofluar 1.25 oil immersion objective lens and a HB100 mercury lamp for epifluorescence was used first. The excitation wavelength of the filter was 450–490 nm, and the emission was detected > 515 nm (filter set 09: BP 450–490, FT 510, LP 515). Digitized pictures were recorded with an AxioCam 305 color CCD camera (Zeiss) using the ZEN 2.3 SP1 Blue software package (Zeiss).

Later on and in order to obtain a better spatial resolution, Airyscan images were acquired in the super resolution mode on a Zeiss LSM 880 microscope (Zeiss, Jena, Germany) equipped with an Airyscan detector using a Plan-Apochromat 63×/1.4 N.A. oil DIC M27 objective. Alexa Fluor 488 was excited with a 488 nm Argon laser line (0.11%) using MBS 488/561/633 and the emission was captured through an SBS LP 460 in combination with emission filter BP 420–480 + BP 495–550 (detector gain 879). Pixel size in Airyscan super resolution mode acquisition was applied automatically in ZEN 2.3 software for the Alexa Fluor 488 Channel, usually resulting in a pixel size of 40 × 40 nm. The Pixel Dwell was 6.1 μsec and the line average was 8. Z-Stack scans were performed all 0.18 μm. DIC images were scanned with a transmitted light T-PMT detector.

### Immunoelectron microscopy (freeze-fracture immunolabeling, FRIL) of cells of *Oxyrrhis marina*

FRIL electron microscopy on freeze-fracture replica obtained from *Oxyrrhis* cells was conducted according to Fujimoto (1997) and Westermann et al. (2005) using the schedule and dilutions described above for the immunoelectron microscopy of proteorhodopsin fraction B3. Thus, the grids with the replica were floated for 1 h onto 50 μl drops of 1% of BSA dissolved in TBS and then incubated for 1 h on 50 μl drops of either 1% of BSA dissolved in TBS or antiserum (diluted 1:100 in TBS containing 0.1% BSA). After washing in 10 drops of TBS and incubation for 1 h in gold-labeled goat anti-rabbit IgG conjugate (diluted 1:50 in TBS containing 1% BSA, 10 nm gold particles), the replica were finally washed with 10 drops of TBS and used for transmission electron microscopy.

## Results

### SDS-PAGE, antiserum production, and western immunoblotting

**Fig. 1:**
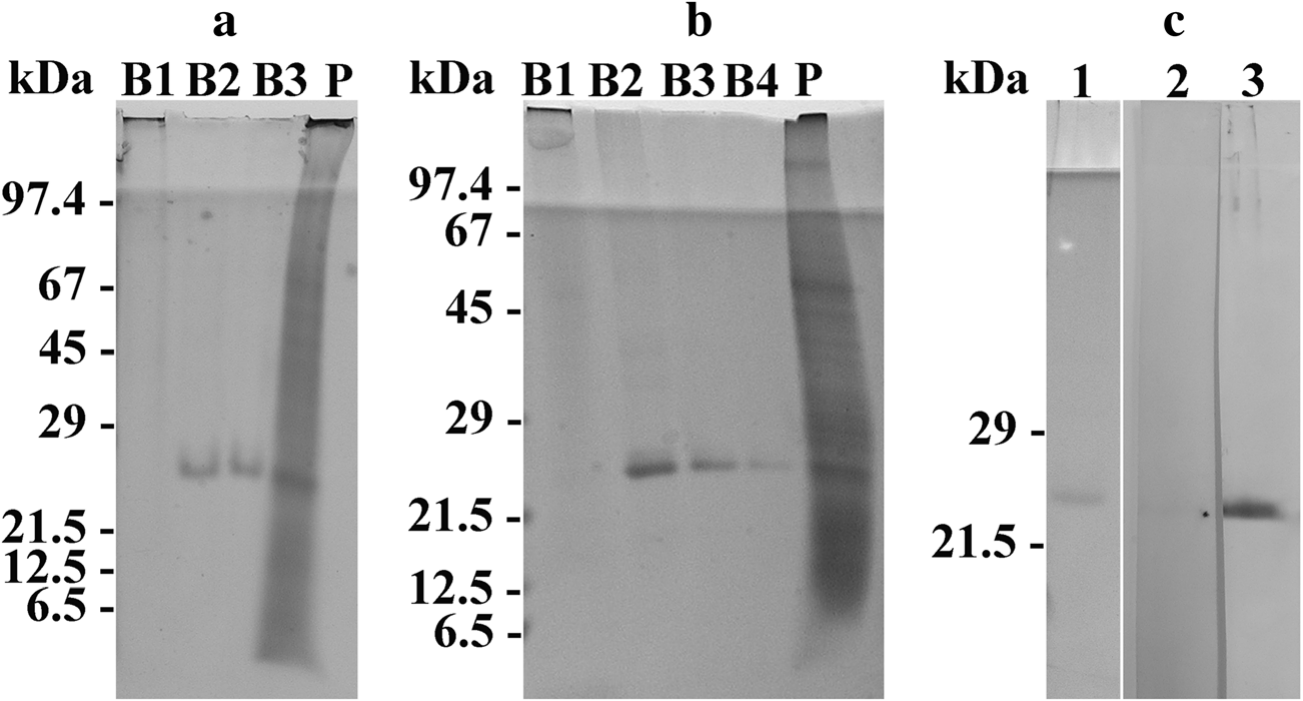
Panels **a** and **b** reveal the protein banding patterns of the sucrose gradient bands (lanes marked B1, B2, B3, B4) and pellet (lane marked P) which were obtained by sucrose density gradient centrifugation and subjected to SDS-PAGE. Note, that a and b show the results obtained from two independent experiments. In the experiment shown in a, three sucrose gradient bands were obtained, whereas four bands were registered in the experiment shown in b. The sucrose gradient bands B2, B3, and B4 show exclusively a protein band of 25 kDa. In panel **c**, the western blots of a pooled proteorhodopsin fraction which had been stained with Ponceau S (lane 1) or immunodecorated either with the preimmune serum (lane 2) or the antiserum directed against the pooled proteorhodopsin fraction (lane 3) are shown. The antiserum labeled proteins of 25 kDa. The relative molecular weights of the marker proteins (kDa) are indicated on the left in a, b, and c. For better display of the bands, the digitized pictures were adjusted for brightness and contrast

Sucrose density gradient centrifugation resulted in three or four red-colored bands and a pellet which consisted of non-solubilized membranes (not shown). SDS-PAGE revealed a multitude of most likely membrane proteins from < 10 up to > 100 kDa for the non-solubilized membrane pellet (Fig. 1a, b, lanes marked P). Band B1 was completely devoid of any protein (Fig. 1a, b, lanes marked B1), and the bands B2, B3, and B4 showed a protein band of 25 kDa (Fig. 1a, b, lanes marked B2, B3, and B4). The bands B2, B3, and B4 were pooled from several experiments, concentrated by centrifugation, and used for SDS-PAGE (Fig. 1c, lane 1) and immunization. Western immunoblotting experiments in which a mixture of B2, B3, and B4 had been subjected to SDS-PAGE revealed strong labeling of proteins of 25 kDa when the antiserum was used (Fig. 1c, lane 3). No immunoreaction was observed using the preimmune serum (Fig. 1c, lane 2).

### Negative staining of bands B2, B3, and B4

**Fig. 2:**
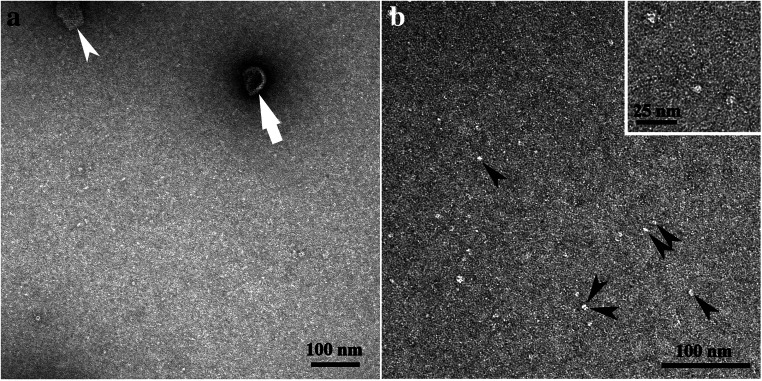
Micrographs of negative-stained band B2, obtained by sucrose density gradient centrifugation. **a** Note the cup-shaped structure (white arrow) and the sheet-like structure (white arrowhead). **b** The monomeric/oligomeric particles are shown in higher magnifications. Some are marked with black arrowheads. The insert in micrograph b is further enlarged. For better display, the digitized pictures were adjusted for brightness and contrast. The scale bars are indicated

**Fig. 3:**
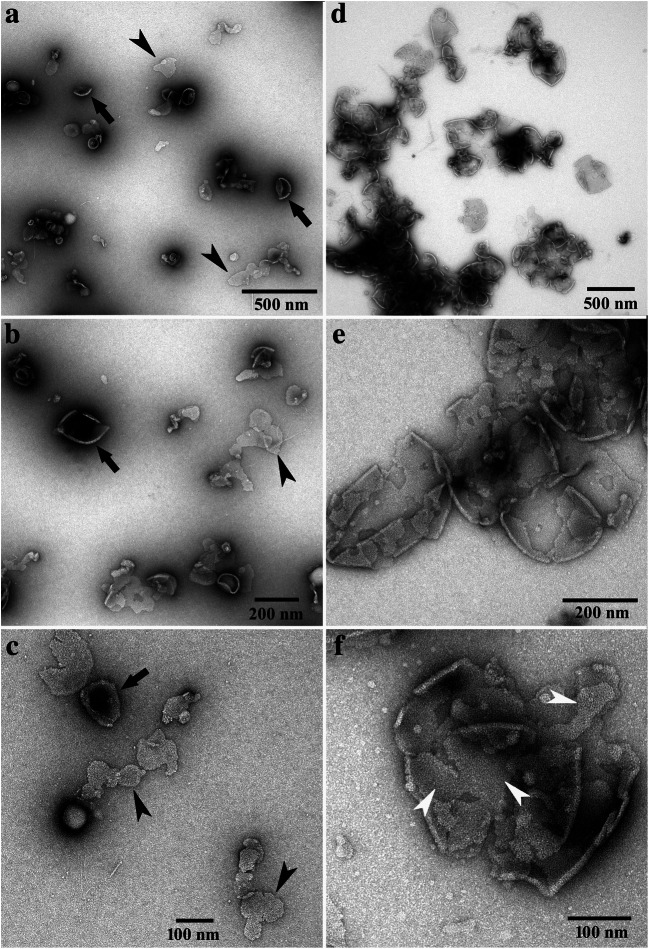
Micrographs of negative-stained bands B3 (micrographs **a**–**c**) and B4 (micrographs **d**–**f**), obtained by sucrose density gradient centrifugation. Note the cup-shaped structures (black arrows) and the sheet-like structures (black arrowheads). Ring-like protein aggregates are indicated by white arrowheads in micrograph f. For better display, the digitized pictures were adjusted for brightness and contrast. The scale bars are indicated

**Fig. 4:**
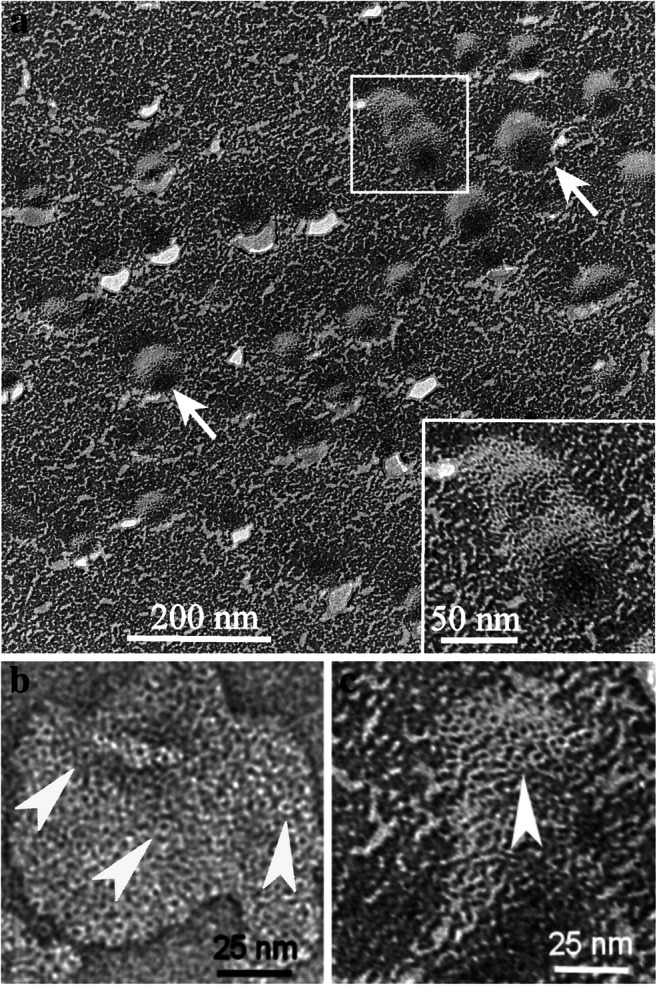
Band 3 imaged by freeze-fracture (micrographs **a** and **c**) and negative staining (micrograph **b**). Note the vesicles (arrows) in the overview and enlarged in the inset of micrograph a and the ring-like aggregates (arrowheads) of proteins in the enlarged images b and c. The dimensions of the ring-like structures were measured in negative-stained samples (micrograph b) with 4.1 ± 0.4 nm in diameter and 1.5 ± 0.2 nm for the hole in the middle. After freeze-fracture preparation (micrograph c), the ring-like structures were measured with 4.0 ± 0.6 nm in diameter and 1.5 ± 0.2 nm for the hole in the middle. For better display, the digitized pictures were adjusted for brightness and contrast. The scale bars are indicated

Band B2 showed minor amounts of sheet-like structures of irregular shape and size (indicated by a white arrowhead in Fig. 2a). Additionally, a few cup-shaped structures were registered (indicated by a white arrow in Fig. 2a). Most of them were rather small-sized, not exceeding 100 nm in diameter. Most likely monomeric/oligomeric particles of 5–6 nm were mainly registered (indicated by black arrowheads in Fig. 2b). In Fig. 3, micrographs obtained by negative staining of the bands B3 (Fig. 3a–c) and B4 (Fig. 3d–f) are compiled. Both bands were dominated by the sheet-like and cup-shaped structures, with band B4 showing larger aggregates of them (Fig. 3d–f). Some of the sheet-like and cup-shaped structures are marked with black arrowheads and arrows, respectively. The rims of the cup-shaped structures were noticeable and gave the impression that the cups represented rather collapsed, dented vesicles (see structures marked with arrows in Fig. 3a–c). Micrographs taken at higher magnification showed that these structures seemed to be composed mainly of protein, and occasionally, ring-like protein aggregates were registered (indicated by white arrowheads in Fig. 3f, and also in Fig. 4b). In negative-stained samples, the ring-like protein aggregates measured 4.1 ± 0.4 nm in diameter, with a central, tiny hole of 1.5 ± 0.2 nm. As bands B3 and B4 showed similar structures, band B3 was further studied by freeze-fracture, Cryo-TEM, and immunoelectron microscopy.

### Freeze-fracture and Cryo-TEM of band B3

**Fig. 5:**
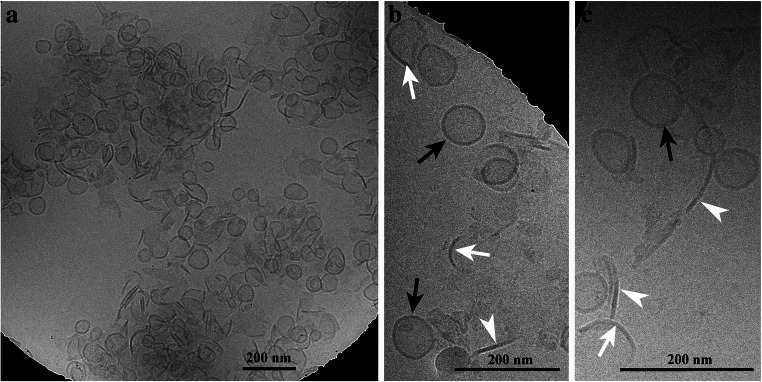
Cryo-TEM micrographs of sucrose gradient band B3 showing vesicular, cup-shaped and sheet-like structures. Note the aggregates in the overview micrograph (**a**) and the vesicles (black arrows), cup-shaped structures (white arrows) and the sheet-like structures (white arrowheads) in (**b**) and (**c**). The shape of vesicles appears in some cases spherical but in most cases it is not totally spherical. For better display, the digitized pictures were adjusted for brightness and contrast. The scale bars are indicated

Micrographs of freeze-fractured cup-shaped structures of band B3 are shown in Fig. 4a and c. Similar to the finding obtained by negative staining (Fig. 4b), ring-like protein aggregates were registered on one occasion, too. In freeze-fractured samples, they measured 4.0 ± 0.6 nm in diameter with a tiny hole of 1.5 ± 0.2 nm in the center (see structures in Fig. 4c, marked with an arrowhead). Thus, they were similar in size to those obtained by negative staining (see Fig. 4b). Using Cryo-TEM, structures similar to those registered by negative staining transmission electron microscopy were observed. The results are compiled in Fig. 5, with Fig. 5a showing an overview micrograph. Cryo-TEM revealed that the cup-shaped structures shown in Fig. 3 were not the result of collapsed vesicles. They were either straight like sheets or slightly bended at the bottom, thus forming the cup. Beside the cup-shaped structures (marked with white arrows in Fig. 5b), vesicles (marked with black arrows in Fig. 5b and c) and the sheet-like structures (marked with white arrowheads in Fig. 5c) were registered, too. The sheets measured 6.5 ± 0.7 nm in thickness and up to 200 nm in length. The vesicles and cup-shaped structures measured in mean 60 ± 12 nm in diameter and similar to the sheets 6.5 ± 0.7 nm in thickness. Occasionally stacked sheets were registered, too.

### Immunoelectron microscopy of band B3

**Fig. 6:**
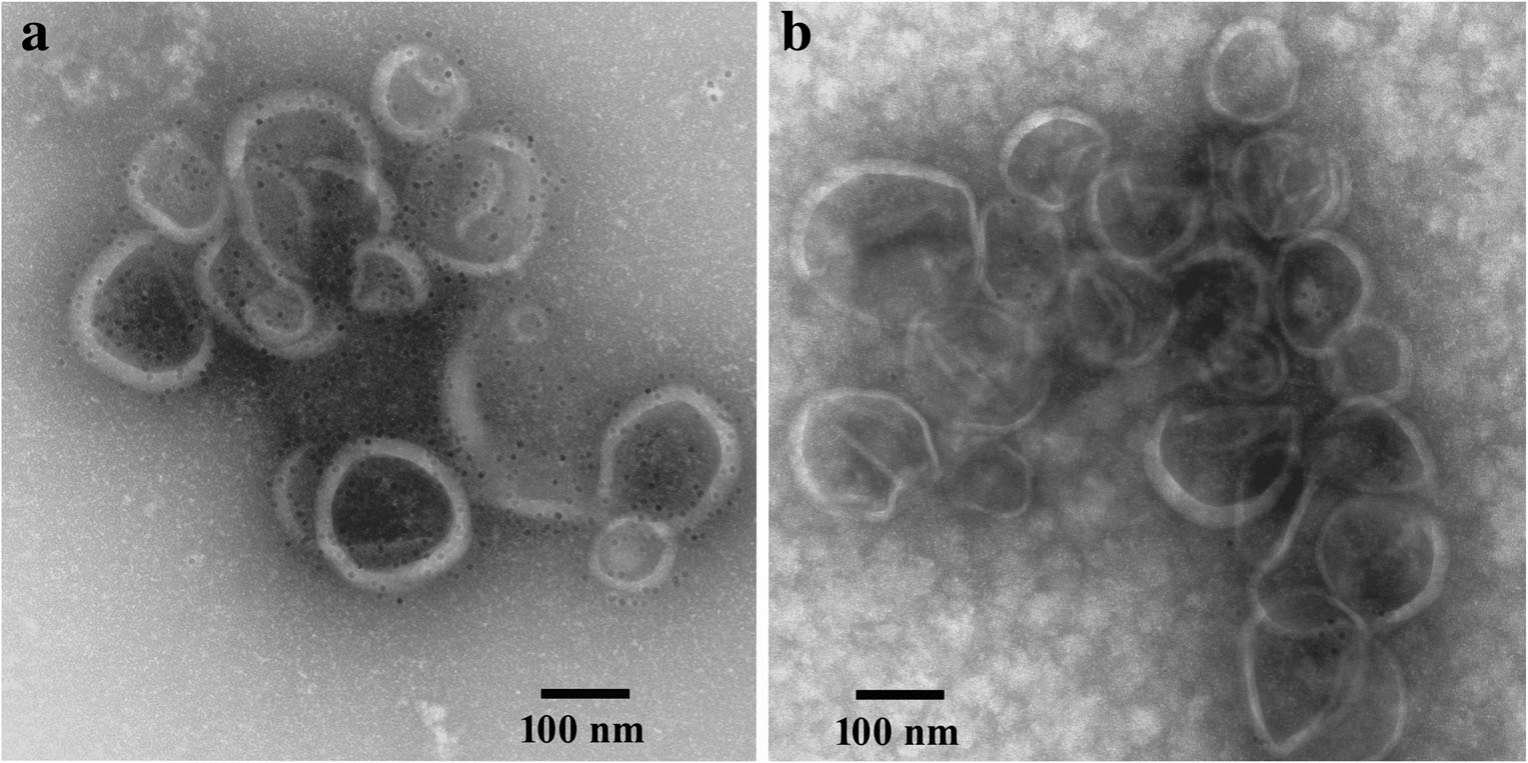
Micrographs of negative-stained band B3. The vesicle-like structures shown in micrograph (**a**) were treated with the antiserum directed against the pooled proteorhodopsin fraction, followed by the second gold-labeled antibody. The control (10 nm gold-labeled goat anti-rabbit IgG conjugate) alone showed no specific binding to the vesicle-like structures (**b**). For better display, the digitized pictures were adjusted for tonal value, brightness, and contrast. The scale bars are indicated

Immunogold labeling followed by negative staining of band B3 resulted in intense labeling of the vesicular, and sheet-like and cup-shaped structures when the antiserum was used (Fig. 6a). Some gold particles were also registered apart from these structures, indicating that either monomeric or oligomeric aggregates of proteorhodopsins were labeled. The application of the second antibody alone did not give rise to significant amounts of gold particles on/at these structures or on the areas in between (Fig. 6b).

### Immunofluorescence light microscopy of *Oxyrrhis* cells

**Fig. 7:**
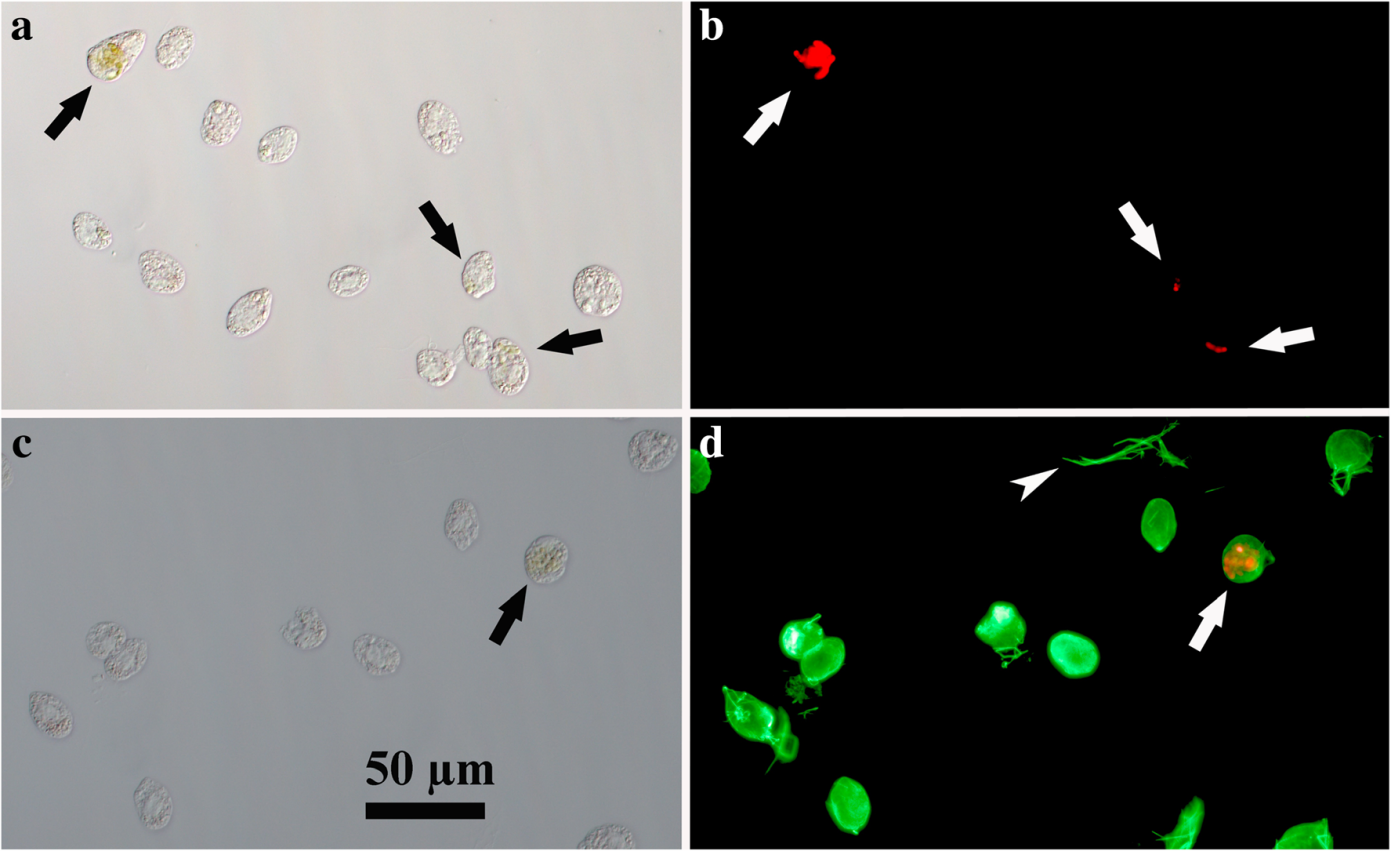
Bright field (**a**, **c**) and Alexa488 immunofluorescence (**b**, **d**) light microscopical micrographs of *Oxyrrhis marina* cells. The binding of the antiserum to the cell periphery is shown in micrograph **d**, with the same area shown in bright field in **c**. The second antibody does not label the cells (micrograph **b**), with the same area shown in bright field in **a**. Note the fluorescence labeling of discharged trichocysts (marked with an arrowhead in **d**), and the red fluorescence of preyed diatom cells (marked with arrows). For better display, the digitized pictures were adjusted for brightness and contrast. A scale bar is indicated in **c**

**Fig. 8:**
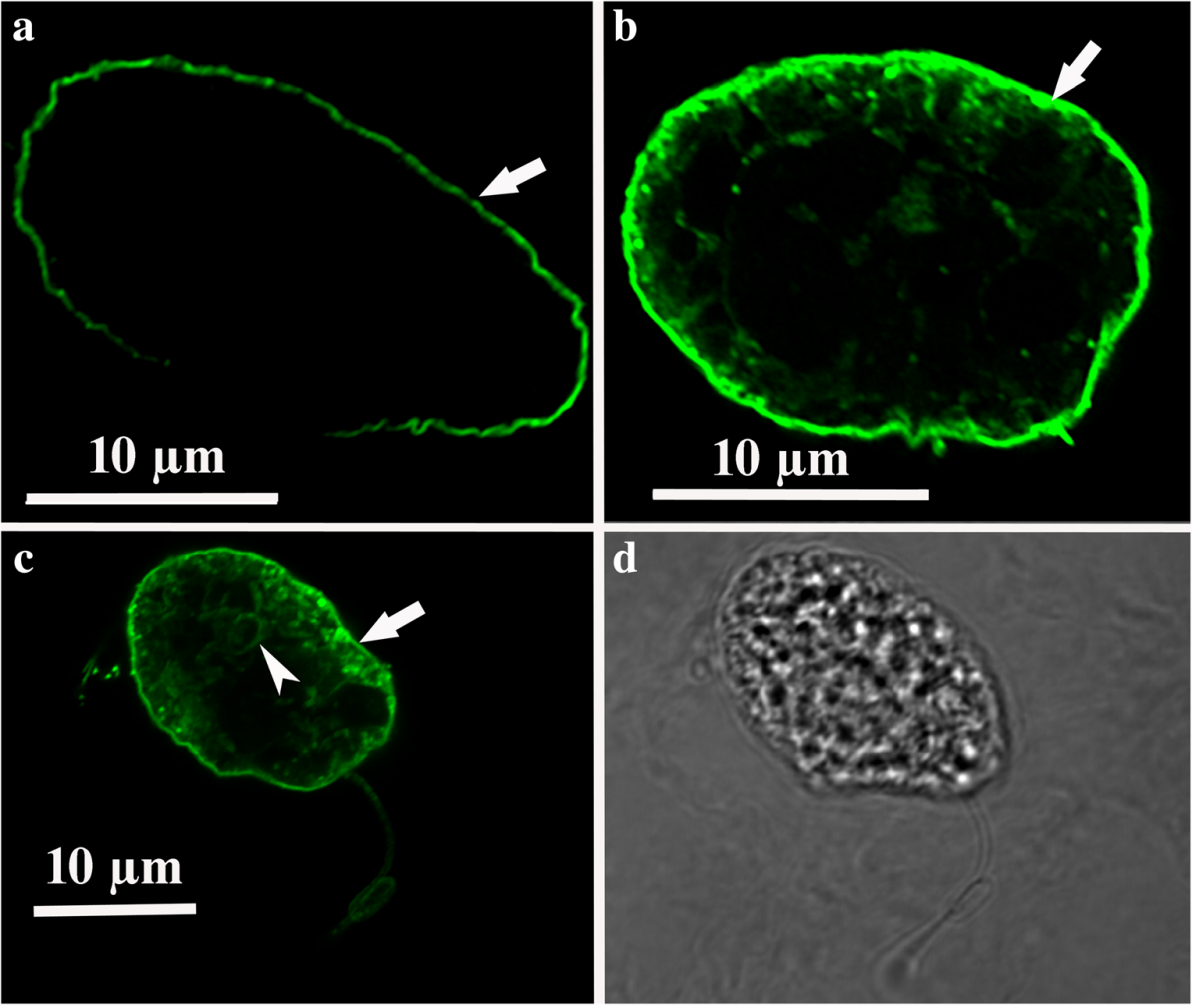
Photographs obtained by CLSM of *Oxyrrhis marina* cells. The binding of the antiserum to the cell periphery is shown in micrographs (**a**), (**b**), and (**c**) (marked with arrows). Note the fluorescence labeling of internal cell components of the cell shown in c (marked with an arrowhead), with the same cell shown in bright field in micrograph (**d**). For better display, the digitized pictures were adjusted for brightness and contrast. Scale bar are indicated in a, b, and c

The results obtained by immunofluorescence light microscopy of *Oxyrrhis* cells are compiled in Figs. 7 and 8. No green immunofluorescence was detected when the Alexa488 labeled antibody was used alone (Fig. 7b), whereas an intense labeling of the cell periphery was registered using the proteorhodopsin antiserum prior to the second antibody (Figs. 7D and 8). Besides the cell periphery, discharged trichocysts (arrowhead in Fig. 7d) and some internal structures became labeled, too. The occasionally registered red fluorescence originated from preyed diatom cells (arrows in Fig. 7). In bright field, those preyed diatom cells showed up as brownish areas within *Oxyrrhis* cells and often were hard to see at all (Fig. 7a and b).

### Freeze-fracture immunolabeling of cells of *Oxyrrhis marina*

**Fig. 9:**
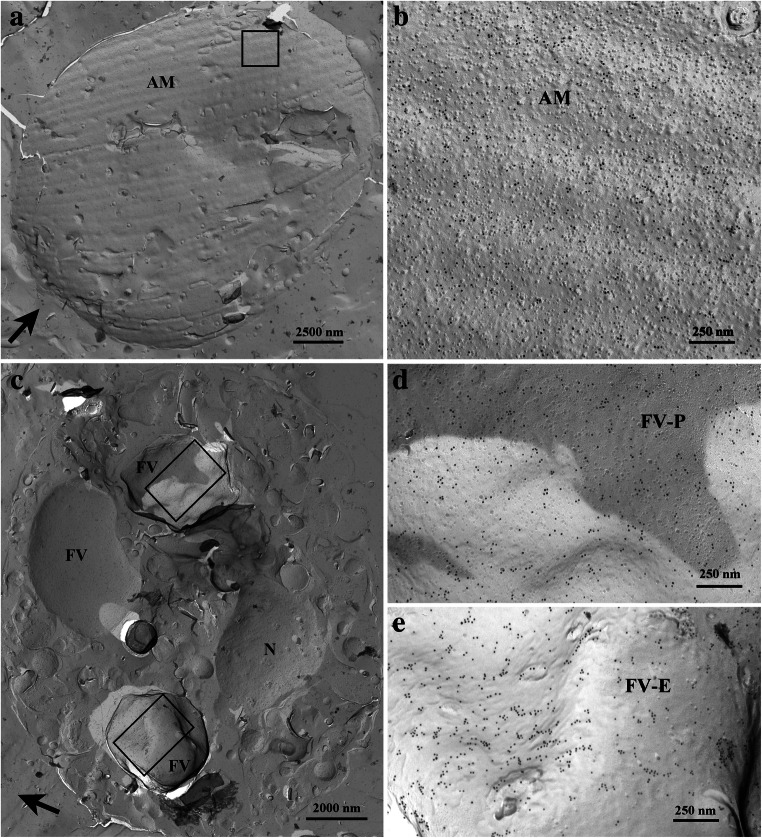
Micrographs of freeze-fracture immunolabeled *Oxyrrhis* cells subjected to electron microscopy. The label of the cell periphery is shown in **a** and **b**. The membranes of the amphiesmal vesicles (AM) became labeled. Within the cells, the membranes of the food vacuoles (FV) became labeled (micrographs **c**, **d**, **e**). Here both, the concave, protoplasmic (FV-P, shown in d) and the convex, exoplasmic fracture faces (FV-E, shown in e) are labeled. In c, the concave fracture of the nuclear membrane (*N*) is unlabeled. The direction of platinum shadowing is indicated by a black arrow in (a, c). For better display, the digitized pictures were adjusted for brightness and contrast. Scale bars are indicated

**Fig. 10:**
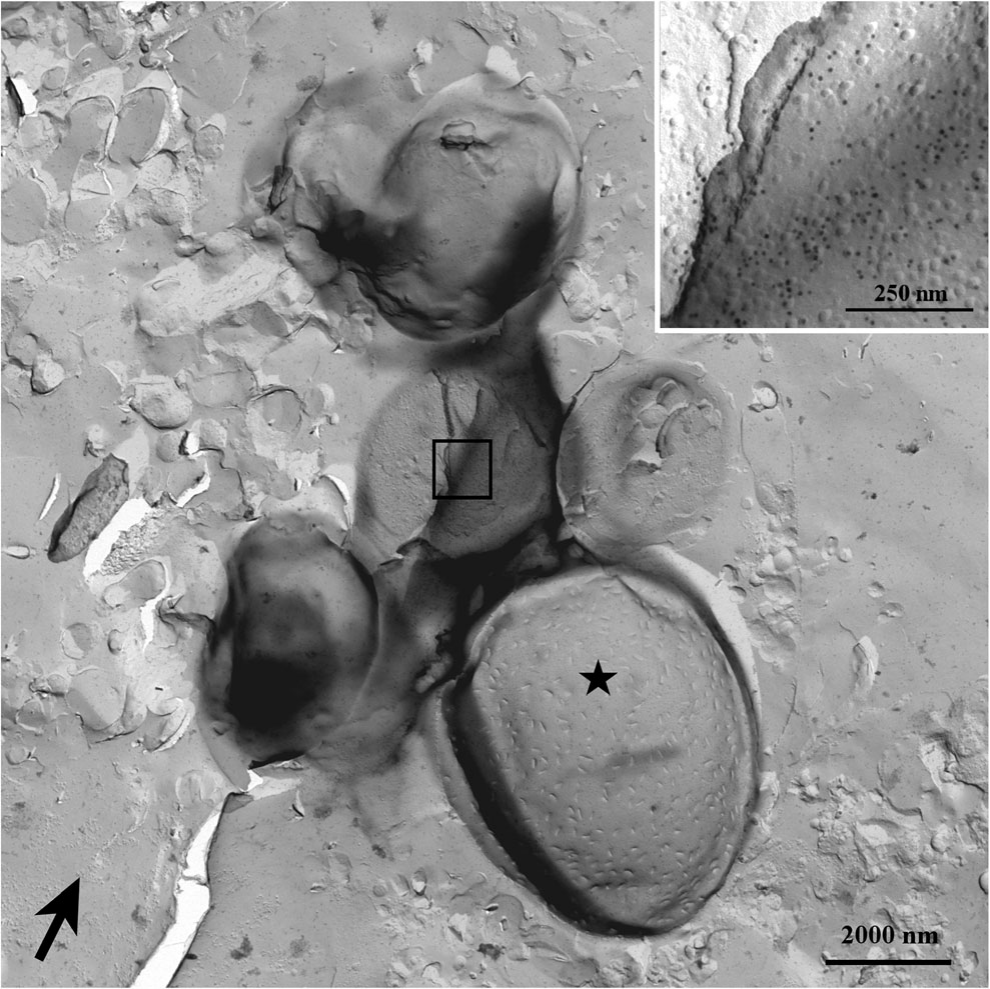
Micrographs of a freeze-fracture immunolabeled *Oxyrrhis* cell subjected to electron microscopy. Remnants of preyed yeast cells mask the label (see insert). The exoplasmic fracture face of the cytoplasmic membrane of a preyed yeast cell can be seen in the lower part (marked with an asterisk). Remnants of the cell wall are still present. The direction of platinum shadowing is indicated by a black arrow. For better display, the digitized pictures were adjusted for brightness and contrast. Scale bars are indicated

CLSM (Fig. 8) showed the binding of the antiserum to the cell periphery and to internal cell components, but did not allow a further discrimination of distinct structures. The cell covering of *Oxyrrhis* comprises the cytoplasmic membrane underlaid by amphiesmal vesicles, whereas several internal organelles are enclosed by membranes. Thus, the immunofluorescence detected at the cell periphery might either be caused by labeling the cytoplasmic membrane, by labeling the amphiesmal vesicles, or by labeling both of them. FRIL was applied in order to elucidate them further. Micrographs obtained by freeze-fracture immunolabeling electron microscopy of *Oxyrrhis* cells are shown in Figs. 9 and 10. Most likely the membranes of the amphiesmal vesicles were labeled at the cell periphery (Fig. 9a and b), while the cell internal label was registered at the food vacuoles (Fig. 9c, d, and e). The membranes of the amphiesmal vesicles showed approximately 2-fold higher amounts of Au-particles per 1 μm^2^ in comparison to other cell compounds, except food vacuoles. For membranes belonging to the food vacuoles, both, P- and E-fracture faces were labeled and showed an approximately 3-fold higher amount of Au-particles per 1 μm^2^ in comparison to other cell internal structures. Remnants derived from preyed yeast cells were often still present on the replica and masked the labeled food vacuoles (Fig. 10). We did not obtain larger areas originating from freeze-fractured cytoplasmic membranes. Therefore, no statements and calculations could be made regarding the labeling intensity of them.

## Discussion

### SDS-PAGE of the bands B2, B3, and B4

The pooled proteorhodopsins present in the sucrose density gradient bands B2, B3, and B4 showed a protein with a molecular weight of 25 kDa when subjected to SDS-PAGE. This result is in line with already published molecular weights determined for proteorhodopsins (Rhiel et al. 2020; Slamovits et al. 2011) and fits to the predicted values of proteorhodopsins of *O. marina*. Rhiel et al. (2020) registered a diffuse protein band immediately above and additional faint bands above and below the 25 kDA protein band. Subsequent MS analyses revealed for them exclusively peptides of proteorhodopsins. These bands were not detected in the current study, most likely due to the fact that less amounts of protein were loaded onto the gels.

### Electron microscopy of the bands B2, B3, and B4

As the band B2 did not migrate deeply into the sucrose gradient, most likely monomeric and/or oligomeric proteorhodopsins seemed to constitute this fraction. The amounts of smaller vesicular and sheet-like and cup-shaped structures were rather low. The sucrose gradient spanned from 30 to 80%, and thus was rather suited for the isolation of larger protein aggregates, membrane fractions, or even cell organelles. This assumption is reflected by the results obtained for bands B3 and B4 which both showed vesicles, membrane-like sheets, and cup-shaped structures which were composed of aggregated proteorhodopsins. Actually, it cannot be stated whether native or artificial proteorhodopsin aggregates were isolated in these two fractions. Triton X-100 might either solubilize larger patches of densely packed, aggregated proteorhodopsins out of the membranes which banded within the gradient, or it might lead to the solubilization into monomers/oligomers which re-aggregate during centrifugation due to the thinning out of the detergent when they migrate into the sucrose gradient. Interestingly, ring-like protein aggregates with diameters of 4.0 nm and showing a tiny hole in the middle were registered with both negative-staining and freeze-fracture transmission electron microscopies. The measured values are similar to those already published for proteorhodopsins. Klyszejko et al. (2008) investigated the oligomeric assembly of proteorhodopsin, overexpressed in *E. coli* and reconstituted into artificial membranes by means of AFM. The protein mainly assembled into crystalline and densely packed regions with hexameric oligomers. A smaller fraction was registered which assembled into pentamers within the non-crystalline areas of the reconstituted membrane patches. The side length of hexagonal lattice measured 8.8 ± 0.7 nm, while the donut-shaped complexes exhibited a diameter of 4.3 ± 0.3 nm. Almost the same value (4.2 nm) was measured for those reconstituted proteorhodopsin fractions using transmission electron microscopy (Shastri et al. 2007).

### Antiserum, immunoelectron microscopy, and immunofluorescence light microscopy

The antiserum raised against the pooled proteorhodopsins present in B2, B3, and B4 nicely immunodecorated the 25 kDa protein band in Western immunoblotting experiments, whereas the preimmune serum did not show any immunolabeling at all. We therefore judged the antiserum to be well suited for immunoelectron microscopy and immunofluorescence light microscopy. The results obtained by immunoelectron microscopy confirmed this assumption, as the structures present in band B3 showed an intense labeling. Subsequent immunofluorescence light microscopy revealed an intense labeling of the cell periphery. This result would be in line with results of Hartz et al. (2011) if the cytoplasmic membrane would have been labeled, because the authors extracted proteins exposed exclusively on the outer plasma membranes of *Oxyrrhis* cells and demonstrated the presence of two rhodopsins (ABV22426 and ABV22427) therein. Ma et al. (2020) investigated the heterologous expression of proteorhodopsins of the dinoflagellates *Prorocentrum donghaiense* and *Alexandrium carterae* in mammalian cells. The authors used a human embryonic kidney cell line (HEK 293 T) for the construction of an expression system for two dinoflagellate proteorhodopsin genes and succeeded in expressing these genes in the system, thus showing that this mammalian cell type was suitable for expressing dinoflagellate genes. Furthermore, the authors registered immunofluorescence of the expressed proteins which locates these dinoflagellate proteorhodopsins on the cell membrane of the mammalian cells. Given the cell peripheral distribution of immunofluorescence in the current study, the cell plasma membrane localization of proteorhodopsins could potentially be the major mode of localization. At the moment, we neither can affirm nor negate the results of Hartz et al. (2011) and Ma et al. (2020), as we did not obtain larger areas of freeze-fractured cytoplasmic membranes which allowed calculation of the amounts of Au-particles per μm^2^. However, immunoelectron microscopy of freeze-fractured cells revealed that membranes of the amphiesmal vesicles were labeled at the cell periphery. It might be that proteorhodopsins occur in both the outer plasma membranes and the membranes of the amphiesmal vesicles. Assuming that the orientation of proteorhodopsins within either the cytoplasmic membrane or the amphiesmal vesicle membranes would be similar, i.e., outward directed, they would still be able to function as light-driven ion (H^+^, Cl^−^, Na^+^) pumps, and, in case they translocate H^+^, the generation of a H^+^ gradient for the synthesis of ATP could be postulated for both. In case other ions become translocated into the thecal vesicles, the proteorhodopsins might function in homeostasis and/or osmoregulation. Some internal structures became labeled in the current study, too. This result fits to observations of Slamovits et al. (2011) who raised an antiserum against a synthetic oligopeptide which represented the deduced C-terminal amino acid sequence of proteorhodopsin OM27 (equivalent to ADY17806). In subsequent immunolabeling light microscopical studies, the authors registered that the proteorhodopsin was not distributed evenly within the cytosol. Ring-like structures were observed in several cells, and no evidence was found that the proteorhodopsin was localized within the plasma membrane, the nucleus, or mitochondria. Therefore, the authors supposed the endomembrane system to harbor proteorhodopsins. Based on the current findings obtained by immunofluorescence light microscopy and immunoelectron microscopy of freeze-fractured cells, we assume that the cell’s internal proteorhodopsins are located mainly within the membranes belonging to the food vacuole. These membranes originally derived from the cytoplasmic membrane, and it would make sense that they harbor proteorhodopsins. In case that the proteorhodopsins would translocate H^+^, they would acidify the interior milieu of the food vacuole upon illumination, thus facilitating digestive enzymes to work properly. In the current study, discharged trichocysts became labeled, too. This finding pinpoints to a contamination of the pooled proteorhodopsin fractions with fragments/proteins of trichocysts and might be explained by a result of Rhiel et al. (2020). Although the authors detected mainly peptides of proteorhodopsins in the 25 kDA protein band, they registered peptides of CAMPEP_0190382616 in one of the MS analyses. Rhiel et al. (2018) assumed that the protein represents a TMP of *O. marina*, as this protein was listed among DELTA-BLAST hits in database deposited EST sequences of *O. marina* when mature trichocyst matrix proteins (TMPs) of the ciliate *Paramecium tetraurelia* were used as query sequences.

In summary, it can be stated that the fractions obtained by sucrose density gradient centrifugation were mainly composed of either monomeric/oligomeric proteorhodopsins (band B2) or aggregates which resemble vesicles, membrane-like sheets, or cup-shaped structures (bands 3 and 4). Micrographs taken at higher magnification pinpoint to ring-like protein aggregates with dimensions described for assembled proteorhodopsin protomers. The antiserum against the proteorhodopsin fraction immunolabeled membranes of the amphiesmal vesicles in the periphery and food vacuole membranes.
